# Survival and Mortality in Hospitalized Children with COVID-19: A Referral Center Experience in Yazd, Iran

**DOI:** 10.1155/2023/5205188

**Published:** 2023-07-17

**Authors:** Farimah Shamsi, Mehran Karimi, Zahra Nafei, Elahe Akbarian

**Affiliations:** ^1^Center for Healthcare Data Modeling, Department of Biostatistics and Epidemiology, School of Public Health, Shahid Sadoughi University of Medical Sciences, Yazd, Iran; ^2^Children Growth Disorder Research Center, Shahid Sadoughi University of Medical Sciences, Yazd, Iran

## Abstract

**Introduction:**

COVID-19 prognostic risk factors, therapeutic protocols, and clinical outcomes in pediatric cases are still under investigation.

**Materials and Methods:**

This historical cohort study evaluated the survival time of hospitalized children (1 month–18 years old) with COVID-19 admitted from March 2020 to August 2021 to an educational hospital in Yazd, Iran. The follow-up of patients was performed at least one month after discharge.

**Results:**

From 183 hospitalized cases, 24 children were deceased. The median age of patients was 5.41, and 54.2% were male. The survival rate after one-month follow-up was 0.88, and the most significant predictors associated with survival time were the male sex, positive history of hospitalization, lymphopenia, hypoxia, and length of stay more than two weeks using Bayesian Cox regression analysis.

**Conclusion:**

Accurate estimation of the impact of predictors on poor outcomes may help healthcare providers use therapeutic protocols based on risk factors and healthcare requirements of each patient to improve their survival.

## 1. Introduction

Coronavirus disease (COVID-19) caused by severe acute respiratory syndrome coronavirus 2 (SARS-Cov-2) started in Wuhan, China, in December 2019 and was stated as a pandemic and public health emergency by the World Health Organization. Iran reported the first death due to COVID-19 among Middle East countries, and a mortality rate of 2% (7, 588, 477 patients and 145, 269 deaths of COVID-19) has been announced in Iran until March 2023 [1]. Although the impact of epidemiological characteristics, laboratory findings, clinical symptoms, and other risk factors in adults was studied through various types of research [2–6], chief complaint, treatment protocols, and prognosis risk factors are not clearly defined in children infected with COVID-19. Moreover, limited studies have explored pediatric cases' survival and long-term clinical symptoms after discharge in this age group. Some studies revealed that children with COVID-19 are mild or asymptomatic [7, 8]; however, there is a lack of information about pediatric severe pulmonary manifestations in new virus variants [9, 10]. In addition, a better understanding of significant chronic complications after COVID-19 infection [11, 12] and the impact of multisystem inflammatory syndrome (MIS-C) on the outcome of infection in children may prevent the increasing mortality rate and the growing treatment costs in the future. Based on available evidence, a few studies estimated survival probabilities regarding risk factors, laboratory findings, and treatments of children with COVID-19 that would be highlighted in this study using different statistical approaches.

## 2. Material and Methods

### 2.1. Study Design and Data Collection

The present paper is written based on the STROBE statement for reporting historical cohort studies [13]. In this research, children aged 1 month–18 years old were admitted consecutively to Shahid Sadoughi COVID-19 referral hospital, including those directly referred to the emergency ward or referred from other hospitals in Yazd province. These patients were diagnosed based on laboratory-confirmed SARS-CoV-2-positive swabs (RT-PCR), clinically confirmed by the clinicians according to defined symptoms or confirmed through CT-scan radiographic findings. The hospitalized patients were enrolled from March 1, 2020, to August 1, 2021, and demographics, underlying diseases, laboratory findings, and treatment protocols were gathered through the hospital information system. Outcomes of patients were recorded through follow-ups for at least one month. The study was approved by the Research Ethics Committee of the Shahid Sadoughi University of Medical Sciences and Health Services, Yazd (IR.SSU.REC.1401.049). Data anonymization was performed before analysis, and the technique of data masking was used for data anonymization in this study. At first step, all the information of each patient was recorded in an excel sheet, and at the second step, the identifying number (defined specific codes for this study) was given to each patient. At the last, step data swapping was performed; patients' privacy and data security were prioritized at all levels, and the study was accomplished under the Helsinki Declaration (2013) guidelines.

### 2.2. Patient's Characteristics, Treatments, and Outcomes

Demographic data (age and sex), underlying diseases, preadmission, on-admission clinical symptoms, length of hospital stay (day), PICU admission, vital signs on admission (respiratory rate, systolic and diastolic blood pressure, and oxygen saturation in an air room (O_2_sat)), patients who suffered from multisystem inflammatory syndrome of children (MIS-C) which was diagnosed based on the WHO criteria, laboratory findings (the first valid value of laboratory parameters was considered), and chest CT-scan findings (pattern and severity score) of 183 patients were collected from medical records. The severity of the disease is defined based on WHO recommendations definition in three categories: nonsevere (absence of signs of severe or critical diseases), severe (O_2_sat lower than 94% on room air, tachypnea, and signs of severe respiratory distress), and critical (requires life-sustaining treatment, acute respiratory distress syndrome, sepsis, and septic shock) [14, 15]. Based on the WHO definition and observed symptoms, the severity of each patient's condition was recorded in the electronic profile. The primary outcome was determined as the death from COVID-19 versus survival after at least one-month follow-up, and the patients who were alive at the last follow-up time were defined as censored.

### 2.3. Statistical Analysis

Continuous and categorical variables were described using the mean ± SD and frequency (percentage). In order to compare the difference of explanatory variables in survived and deceased groups, the Pearson chi-square and Fisher exact tests were used. Cramer's V and Eta were considered as the measure of association between independent variables and outcome. The Kaplan–Meier method and the log-rank test were performed to estimate and compare the survival functions in different categories of each explanatory variable. Using a stepwise process, the Cox proportional hazards model was fitted, including significant independent variables in univariate analysis. In the multivariate approach, Bayesian survival analysis was conducted to estimate significant independent variables to overcome a few outcomes. The survival analysis was conducted using RStudio (RStudio Team (2020), RStudio: integrated development for R. RStudio, PBC, Boston, MA URL https://www.rstudio.com/) and packages survival, KMsurv, spBayesSurv, and coda. The significance level of 0.05 was regarded as statistically significant.

## 3. Results

### 3.1. Characteristics, Clinical Findings, and Outcome of Children with COVID-19

A total of 183 children aged 1mo–18 y were included in this study; 54.2% and 45.8% were males and females, respectively, with a median age of 5.41 (mean= 6.95 and standard deviation = 5.59). Thirteen percent (*n* = 24) of children with COVID-19 died that most aged 1–5; however, the association between the age groups and death was insignificant. The previous hospitalization, PICU admission, abnormal long CT findings, O_2_sat, patient intubation, ventilator support, and length of hospital stay were significant among all variables. The strength of the relationship between death and variables, including ventilatory support (Cramer's V = 0.52), PICU admission (Cramer's V = 0.49), and patient intubation (Cramer's V = 0.48), was the highest (Table 1). As shown in Table 1, the main preadmission symptoms were fever, cough, dyspnea, and vomiting. On-admission time, fever, and cough were observed among sixty-nine and thirty-four patients, and cough was significantly higher in deceased children. Neurologic diseases were the frequent underlying disease, and MIS-C was observed in 24.6 percent of children. Moreover, 12.6 percent of patients were critical, and as expected, most deceased children were in the severe and critical groups.

### 3.2. Laboratory Finding Data

Table 2 compares the range of laboratory findings between deceased and surviving patients. According to the age of patients, increased AST, CPK, and lymphopenia showed marginal significance.

### 3.3. Drugs Used for Patients

In this study, 28 children received methylprednisolone (21 in survived and 7 in deceased groups), and the differences between the two groups were significantly meaningful (*P* = 0.04). Fifty-three children received remdesivir, and the death rate was 33%, showing no significant difference between the two groups. In addition, only 10.5 percent of children received favipiravir, and all survived. No significant difference in the mortality rate was observed among other drugs used for patients (Table 3).

### 3.4. Survival Rate of Children with COVID-19

**Figure 1:**
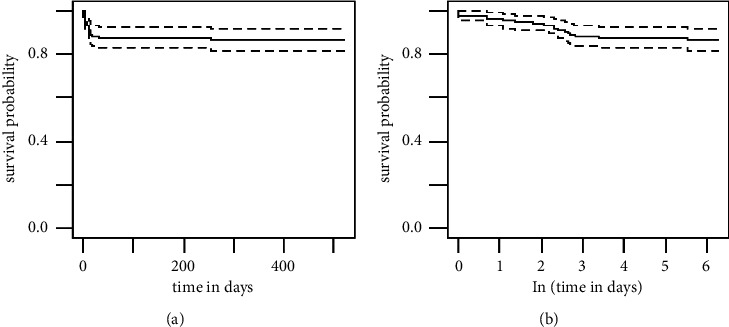
(a, b) The Kaplan–Meier survival time and Ln (survival time) of children with COVID-19 in Shahid Sadoughi Hospital in Yazd.

**Figure 2:**
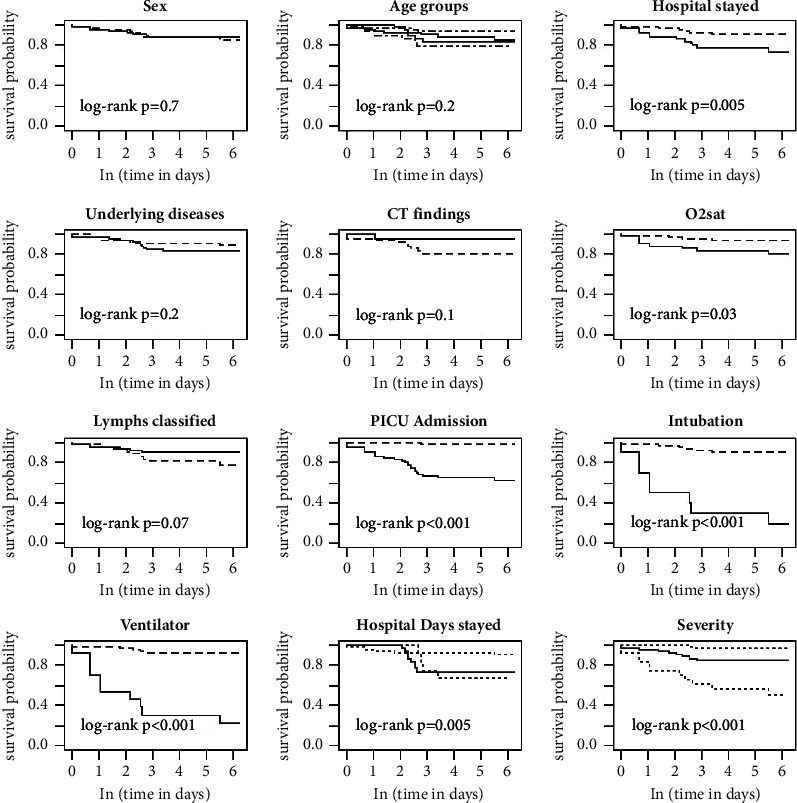
The Kaplan–Meier Ln (survival time) by explanatory variables of children with COVID-19 in Shahid Sadoughi Hospital in Yazd.

The survival rate of hospitalized children and their risk factors were evaluated by Kaplan–Meier estimates (Figure 1(a)). The overall survival rates of patients on the first and 10^th^ and 30^th^ days of follow-up were 0.98, 0.91, and 0.88, respectively. The natural logarithm of the follow-up time was used for the other analysis to present the survival rate differences among risk factors more precisely (Figure 1(b)). Based on the log-rank test, the variables significantly increased the death rate were length of hospital stay, the severity of disease, positive history of previous hospitalization, low O_2_sat on admission, PICU admission, patient intubation, and ventilatory support, respectively (Figure 2). According to the age of the patients, lymphopenia had a slight impact on the survival rate. The hazard ratio of each significant variable was assessed using univariate and multivariate Cox regression. In univariate analysis, as expected, most of the children admitted to the PICU died, and the hazard ratio of death was 16 times more in patients with ventilatory support.

Furthermore, the hazard ratio of death was 14 times more in intubated patients. The recent history of hospitalization, hypoxia (O_2_sat lower than 94%), elevated CPK value above 200 (U/L), and duration of hospitalization of more than one week could increase the risk of death by more than threefold. As shown in Table 4, based on the backward method of Cox multivariate analysis, none of the variables showed a considerable impact on the risk of death. The multivariate Cox regression failed to estimate the actual effect of covariates because of a few samples in some categories of predictive variables.

### 3.5. The Bayesian Hazard Estimation of Variables

Bayesian survival analysis was regarded as a practical statistical approach for predicting survival rates in such data where the occurrence of outcome was not high. As mentioned in the result section, only 13% of hospitalized children with COVID-19 died, and the patients' overall survival rate on the 10^th^ day of follow-up was higher than 90%. In addition, some diagnostic procedures, such as CT scans or some treatments, including intubation and ventilatory support, were not used for many children. As a result, the actual estimate of covariates in the survival rate prediction could not be calculated. In the last step of the analysis, the Bayesian Cox regression was conducted based on normal priors for the coefficient's parameters. Trace diagrams were considered the measures of model assessment. All trace diagrams were converged, and the coefficients of variables are presented in Table 5. As shown, the risk of death was 50% higher for boys; however, the survival rate of age groups was not significantly different. The risk of death for children with a positive history of hospitalization diseases and underlying diseases would increase to 2.77 and 1.75, respectively. In children with O_2_sat lower than 94%, death occurred 2.3 times more than in patients with normal O_2_sat, and lymphopenia patients had a higher risk of death (HR = 1.27%, 95%CI: 1.18–1.36). In addition, the hospitalization duration of more than two weeks would increase the hazard of death to 89% for children.

## 4. Discussion

Since the global research on mortality and survival in children with COVID-19 was insufficient or the data collected were limited, this study was undertaken to record and report a large subset of relevant variables. Moreover, in this research, each patient was followed at least one month after discharge, which was not investigated in other studies. According to the data, 183 patients aged between 1 month and 18 years old were admitted to Shahid Sadoughi Hospital in Yazd between March 2020 and August 2021. The findings of this study included detailed demographics, clinical characteristics, paraclinical data, medications, and their association with survival probabilities. The mortality rate of hospitalized children with COVID-19 was reported as 5.3% in Iran and tripled in children with underlying diseases [16]. In this study, 13% of patients died, two times higher than the overall mortality rate in similar studies in Iran. The mortality rate of 18 years old or younger children with COVID-19 in Europe up to April 2020 was reported as 0.69% and 2.2% in two other studies [8, 17, 18].

Similarly, Oliveira et al. found that the mortality rate in children younger than 20 was 7.8% (15). The death rate could vary in different studies based on substantial differences among countries in epidemiological features, healthcare system, access to standard treatments, and length of follow-up. From studies in China and a meta-analysis in Taiwan, the majority of children with COVID-19 (more than 90%) were reported in mild to moderate status, significantly higher than the observed rate of this research [7, 19]. Shahid Sadoughi Hospital was a referral center for severe patients of COVID-19, and half of the admitted children with COVID-19 were in severe or critical status, which caused a high death rate in this group of patients. Moreover, at the beginning of the COVID-19 epidemic period, physicians' knowledge about the standard treatments for this infection was limited, and only 30% and 15% of patients were treated by Rem and Meth, respectively.

### 4.1. Demographic Distribution

In this study, children aged between 5 and 13 years old suffered more from COVID-19 (33%), which was similar to the results of other studies of Dong et al., Mahmoudi et al., and Swan et al. such as the Center for Disease Control report (CDC report) in the United States [19–22]. Compared with other age groups, children aged between 5 and 13 years old were at a lower risk of death, consistent with the Armin et al. study [23]. No association was found between gender and the death rate in this study. However, slightly more deaths occurred in boys, consistent with other studies [8, 24]. In this research, male children were at a higher risk of severe disease, as in the review of Gallo Marin et al. [25], but the male gender did not seem to be an independent risk factor for severe COVID-19. 27% of children had a positive history of hospitalization, which caused a considerable increase in the death rate. In Iran, the frequency of having at least one underlying disease in patients younger than 18 years old was reported from 8% to 50%, with a death rate of 15% [16, 23]. The data showed a death rate of 45.5% for children with a underlying disease, which was in line with the results of the United States CDC report (42.3%) but significantly higher than the findings of a Brazilian study (28%) and a multicenter cohort study of European pediatric patients (25%) [8, 18, 26]. In some studies, respiratory and neurological diseases were reported as the most common comorbidities in deceased cases, but this study showed malignancies of neurological diseases as the common underlying diseases among children who died due to COVID-19 [27, 28].

Furthermore, previous studies represented a significantly higher mortality rate in hospitalized children with comorbidities, which was not found in this study. In this study, 24% of the patients were complicated by MIS-C, which was higher than in similar studies [29]. As mentioned, half of the admitted patients were severe, which could cause critical outcomes.

### 4.2. Symptoms

Most studies reported fever and cough as the frequent symptoms among hospitalized children with COVID-19. Dyspnea and chest pain were declared as the third and fourth most frequent symptoms in children with severe disease who were admitted to Children's Hospital in New York City, but no significant difference was observed in the frequency of these symptoms in comparison with patients without a severe disease [30]. Similar to a study in Vietnam, dyspnea on-admission time had a statistically significant association with the death rate [31]. In this study, vomiting and diarrhea were frequent among hospitalized children on preadmission or on admission to the hospital. However, any of these symptoms could not be considered a risk factor for death in patients aged <18 years old [32, 33]. O_2_sat lower than 95% was the presenting symptom in 41% of hospitalized Brazilian children, but its association with the mortality rate was not investigated [18]. Based on the findings of this research and other studies, O_2_sat lower than 94% was common among infected children, and there was a negative association between death frequency and oxygen saturation percent [8, 18, 34].

### 4.3. Laboratory Findings and Drugs Used

The result showed that the mortality rate in patients with high AST (U/L) and CPK (U/L) and children with lymphopenia would increase considerably. According to one systematic review published in 2020, hypoalbuminemia, lymphopenia, leukocytosis, elevated interleukin level 6, and prolonged PT time were effective laboratory features associated with the mortality rate in children with COVID-19 [35]. Like this study, a cohort study in Korea presented that lymphopenia and its severity could be considered prognostic variables for clinical outcomes, such as mortality, need for intensive care, and need for intubation and ventilatory support [36].

### 4.4. Survival and Mortality

In this research, the significant risk factors of death related to COVID-19 were a positive history of hospitalization, hypoxia, lymphopenia, elevated serum levels of AST and CPK, need for intubation and ventilatory support, and length of hospital stay of more than one week. Compared with studies of Oliveira et al., Gӧtzinger et al., and Swann et al., there was a weak relationship between underlying diseases and mortality risk [8, 18, 22]. The actual effect of each risk factor was not evaluated, considering the survival of hospitalized pediatric patients. The reason for not reporting the Cox regression analysis result could be a few samples in different categories of predictive variables, which were adjusted using the Bayesian Cox approach in this investigation. Considering the Bayesian Cox regression, patients aged between 1 and 13 years old compared to patients older than 13 years and children without any underlying diseases had a significantly lower risk of death, consistent with previous reports [8, 18, 22]. In addition, lymphopenia showed a significant effect on the survival of pediatric patients. Gallo Marin et al. revealed that O_2_sat <90.5% seemed to be a reliable predictor for patient survival which was not found in this investigation [25].

The most feature of this study was the estimation of the survival rate of COVID-19 inpatient children and its association with predictive factors through two different statistical approaches. Also, this study had two other strengths. First, all of the important variables related to the mortality of COVID-19 patients were recorded, and the survival rate based on the different categories of these variables was evaluated. Second, the children were followed precisely at least one month after discharge. However, some important limitations of the current study were the small population of patients in a single center. In addition, the data were collected from the electronic records of the patients, which had some missing information that affected the outcomes of the study.

## 5. Conclusions

Since SARS-CoV-2 is a novel virus and the number of infected children is not limited, this study provided a proper statistical method to evaluate the association of demographical and clinical profiles and laboratory findings with the survival of hospitalized children. The findings suggested that specific subgroups of children, including boys and those with a positive history of hospitalization and underlying diseases, are at higher mortality risk. The presence of lymphopenia and hypoxia also significantly increases the mortality risk in children with COVID-19. In addition, hospitalization for more than two weeks increases the risk of developing complications from COVID-19 and even death. These results underscore the need for improved therapeutic protocols to control hospital mortality rates. It is recommended that physician pay more attention to clinical and paraclinical risk factors which could increase the severity and mortality in pediatric patients.

## Figures and Tables

**Table 1:** Characteristics, clinical symptoms, and outcomes of children with COVID- 19 in Shahid Sadoughi Hospital in Yazd.

Variables			Total (*n* = 183)	Survived (*n* = 159)	Deceased (*n* = 24)	Cramer's V/Eta	*P* value
Age	Median (IQR) mean (SD)		5.41 (9.42)	5.75 (9.09)	3.20 (11.85)	0.15	0.23
			6.95 (5.59)	7.08 (5.56)	6.1 (5.83)		
	1 month–1 year		28.0 (15.30)	22.0 (13.8)	6.0 (25)		
	1 year–5 years		59.0 (32.2)	51.0 (31.2)	8.0 (33.3)		
	5 years–13 years		60.0 (32.8)	56.0 (32.2)	4.0 (16.7)		
	13 years–18 years		36.0 (19.7)	30.0 (18.9)	6.0 (25)		

Sex	Boys		92.0 (50.3)	79.0 (49.7)	13.0 (54.2)	0.03	0.68
	Girls		91.0 (49.7)	80.0 (50.3)	11.0 (45.8)		

History of hospitalization	Yes		49.0 (26.8)	37.0 (23.3)	12.0 (50.0)	0.20	0.006
	No		134.0 (73.2)	111.0 (76.7)	12.0 (50.0)		

Fever	Preadmission		125.0 (67.7)	108.0 (76.6)	17.0 (77.3)	0.005	0.94
	On admission		119.0 (68.8)	101.0 (66.9)	18.0 (81.8)	0.107	0.16

Cough	Preadmission		62.0 (46.3)	55.0 (46.2)	7.0 (46.7)	0.003	0.97
	On admission		59.0 (34.1)	55.0 (36.4)	4.0 (18.2)	0.13	0.09

Vomiting	Preadmission		56.0 (40.3)	46.0 (38)	10.0 (55.6)	0.12	0.16
	On admission		46.0 (26.6)	37.0 (24.5)	9.0 (40.9)	0.12	0.10

Diarrhea	Preadmission		54.0 (38.6)	48.0 (38.7)	6.0 (37.5)	0.008	0.92
	On admission		47.0 (27.2)	40.0 (26.5)	7.0 (31.8)	0.04	0.60

Dyspnea	Preadmission		53.0 (47.7)	47.0 (47)	6.0 (54.5)	0.045	0.63
	On admission		41.0 (23.7)	32.0 (21.2)	9.0 (40.9)	0.15	0.04

Fatigue	Preadmission		43.0 (23.5)	38.0 (23.9)	5.0 (20.8)	0.02	0.74
	On admission		2.0 (1.1)	2.0 (1.3)	0.0 (0.0)	0.042	0.58

Poor feeding	Preadmission		38.0 (20.8)	35.0 (22)	3.0 (12.5)	0.08	0.28
	On admission		17.0 (9.9)	14.0 (9.3)	3.0 (13.6)	0.048	0.53

Headache	Preadmission		25.0 (13.7)	25.0 (15.7)	0.0 (0.0)	0.15	0.04
	On admission		16.0 (9.3)	15.0 (10.0)	1.0 (4.5)	0.063	0.41

Seizure	Preadmission		21.0 (11.5)	19.0 (11.9)	2.0 (8.3)	0.038	0.60
	On admission		19.0 (10.4)	17.0 (10.8)	2.0 (8.3)	0.027	0.72

Agitation	Preadmission		19.0 (10.4)	16.0 (10.1)	3.0 (12.5)	0.027	0.71
	On admission		7.0 (4.0)	5.0 (3.3)	2.0 (8.7)	0.093	0.22

Extremity pain/muscle pain	Preadmission		28.0 (15.3)	27.0 (17.0)	1.0 (4.2)	0.12	0.10
	On admission		20.0 (11.4)	19.0 (12.5)	1.0 (4.2)	0.09	0.23

Sore throat	Preadmission		11.0 (16.2)	11.0 (18.3)	0.0 (0.0)	0.16	0.19
	On admission		12.0 (6.9)	12.0 (7.9)	0.0 (0.0)	0.10	0.17

Respiratory rate	1 month–1 year	<51	—	—		—	—
		≥51	12.0 (100)	9 (100)	3 (100)		
	1 year–5 years	<30	2.0 (5.4)	2.0 (6.3)	0.0 (0)	0.09	1.00
		≥30	35.0 (94.6)	30.0 (93.8)	5.0 (100)		
	5 years–13 years	<25	7.0 (17.5)	7.0 (19.4)	0.0 (0)	0.15	0.23
		≥25	33.0 (82.5)	29.0 (80.6)	4.0 (100)		
	13 years–18 years	<20	6.0 (40.0)	6.0 (50.0)	0.0 (0)	0.4	0.12
		≥20	9.0 (60.0)	6.0 (50.0)	3.0 (100)		

Systolic/diastolic blood pressure	1 month–1 year	≤105/66	20.0 (100)	16.0 (100)	4.0 (100)	—	—
		>105/66	0.0 (0)	0.0 (0)	0.0 (0)		
	1 year–5 years	≤112/61	47.0 (88.7)	43.0 (93.5)	4.0 (57.0)	0.4	0.02
		>112/61	6.0 (11.3)	3.0 (6.5)	3.0 (43.0)		
	5 years–13 years	≤128/88	48.0 (84.0)	44.0 (83.0)	4.0 (100)	0.12	1.00
		>128/88	9.0 (16.0)	9.0 (17.0)	0.0 (0)		
	13 years–18 years	≤120/80	31.0 (100)	25.0 (100)	6.0 (100)	—	—
		>120/80	0.0 (0)	0.0 (0)	0.0 (0)		

PICU admission	Yes		60.0 (32.8)	38.0 (23.9)	22.0 (91.7)	0.49	<0.001
	No		123.0 (67.2)	121.0 (76.1)	2.0 (8.3)		

Underlying diseases	Yes		81.0 (45.5)	67.0 (42.0)	14.0 (58.5)	0.11	0.14
	No		102.0 (54.5)	92.0 (58.0)	10.0 (41.5)		

Underlying diseases (types)	Neurologic disease		24.0 (13.11)	21.0 (13.2)	3.0 (12.5)	0.24	0.72
	Malignancies		15.0 (8.2)	10.0 (6.3)	5.0 (20.8)		
	Hematologic diseases		12.0 (6.5)	11.0 (7.0)	1.0 (4.2)		
	Cardiovascular diseases		7.0 (3.82)	6.0 (3.8)	1.0 (4.2)		
	Nephrological diseases		6.0 (25.0)	4.0 (2.5)	2.0 (8.33)		
	Respiratory diseases		5.0 (2.73)	4.0 (2.5)	1.0 (4.16)		
	Genetic diseases		5.0 (2.73)	4.0 (2.5)	1.0 (4.16)		
	Gastrointestinal diseases		4.0 (2.18)	3.0 (1.9)	1.0 (4.16)		

CT findings	Normal		19.0 (23.2)	19.0 (27.5)	0.0 (0.0)	0.24	0.03
	Abnormal		63.0 (76.8)	50.0 (72.5)	13.0 (100.0)		

O_2_ saturation	≥94%		92.0 (68.7)	86.0 (71.7)	6.0 (42.9)	0.19	0.03
	<94%		42.0 (31.3)	34.0 (28.3)	8.0 (57.1)		

Intubation	Yes		10.0 (5.5)	2.0 (1.3)	8.0 (33.3)	0.48	<0.001
	No		173.0 (94.5)	157.0 (98.7)	16.0 (66.7)		

Ventilator support	Yes		13.0 (7.1)	3.0 (1.9)	10.0 (41.7)	0.52	<0.001
	No		170.0 (92.9)	156.0 (98.1)	14.0 (83.3)		

MIS-C	Yes		45.0 (24.6)	36.0 (22.6)	9.0 (37.5)	0.12	0.11
	No		138.0 (75.4)	123.0 (77.4)	15.0 (62.5)		

Length of hospital stay (day)	≤7		141.0 (77.0)	129.0 (81.1)	12.0 (50.0)	0.25	0.003
	8–14		30.0 (16.4)	22.0 (13.8)	8.0 (33.3)		
	≥15		12.0 (6.6)	8.0 (5.0)	4.0 (16.7)		

Severity	Not severe		94.0 (51.4)	91.0 (57.2)	3.0 (12.5)	0.42	<0.001
	Severe		66.0 (36.1)	56.0 (35.2)	10.0 (41.7)		
	Critical		23.0 (12.6)	12.0 (7.5)	11.0 (45.8)		

Frequency (%) was used for describing the categorical variables. The Cramer's V, chi-square, and Fisher exact tests were used to measure the association between categorical variables and status.

**Table 2:** Laboratory findings of children with COVID-19 in Shahid Sadoughi Hospital in Yazd.

Variables		Total (*n* = 183)	Survived (*n* = 159)	Deceased (*n* = 24)	Cramer's V/Eta	*P* value
PCR outcomes	Positive	128.0 (70.0)	113.0 (71.0)	15.0 (62.5)	0.06	0.39
	Negative	55.0 (30.0)	46.0 (29.0)	9.0 (37.5)		

WBC (×10^3^/*μ*L)	<5000	104.0 (60.8)	89.0 (60.0)	15.0 (65.2)	0.09	0.47
	5000–15000	37.0 (21.6)	31.0 (21.0)	6.0 (26.0)		
	>15000	30.0 (17.5)	28.0 (19.0)	2.0 (8.8)		

PLT (×10^3^/*μ*L)	<15000	113.0 (67.0)	98.0 (67.6)	15.0 (62.5)	0.05	0.81
	15000–45000	34.0 (20.0)	28.0 (19.3)	6.0 (25.0)		
	>45000	22.0 (13.0)	19.0 (13.1)	3.0 (12.5)		

Lymphopenia	No	105.0 (71.0)	95.0 (73.5)	10.0 (52.5)	0.15	0.06
	Yes	43.0 (29.0)	34.0 (26.5)	9.0 (47.5)		

CRP	0	49.0 (33.0)	43.0 (33.5)	6.0 (33.3)	0.18	0.17
	1+	32.0 (22.0)	26.0 (20.0)	6.0 (33.3)		
	2+	41.0 (28.0)	35.0 (27.0)	6.0 (33.3)		
	3+	25.0 (17.0)	25.0 (19.5)	0 (0.0)		

BUN (mg/dL)	≤20	32.0 (31.7)	26.0 (31.3)	6.0 (33.3)	0.02	0.86
	>20	69.0 (68.3)	57.0 (68.7)	12.0 (66.7)		

CR (mg/dL)	≤1.5	129.0 (83.2)	112.0 (83.0)	17.0 (85.0)	0.02	0.82
	>1.5	26.0 (16.8)	23.0 (17.0)	3.0 (15.0)		

NA (mEq/L)	<135	95.0 (60.5)	84.0 (62.0)	11.0 (52.5)	0.08	0.62
	135–145	58.0 (37.0)	49.0 (36.0)	9.0 (43.0)		
	>145	4.0 (2.5)	3.0 (2.0)	1.0 (4.5)		

*K* (mEq/L)	<3.5	133.0 (88.0)	115.0 (87.8)	18.0 (90.0)	0.07	0.66
	3.5–5.5	13.0 (8.7)	11.0 (8.5)	2.0 (10.0)		
	>5.5	5.0 (3.3)	5.0 (3.7)	0.0 (0.0)		

CA (mg/dL)	<8.8	43.0 (49.5)	38.0 (54.3)	5.0 (29.5)	0.21	0.14
	8.8–10.8	43.0 (49.5)	31.0 (44.3)	12.0 (70.5)		
	>10.8	1.0 (1.0)	1.0 (1.4)	0.0 (0.0)		

MG (mEq/L)	>105	61.0 (97.0)	45.0 (95.7)	16.0 (100.0)	0.10	1.00
	<105	2.0 (3.0)	2.0 (4.3)	0.0 (0.0)		

*P* (mg/dL)	<3	42.0 (82.5)	33.0 (84.5)	9.0 (75.0)	0.14	0.61
	3–6	7.0 (13.5)	5.0 (13.0)	2.0 (16.5)		
	>6	2.0 (4.0)	1.0 (2.5)	1.0 (8.5)		

ALT (U/L)	8–35	65.0 (59.5)	56.0 (60.0)	9.0 (56.3)	0.03	0.76
	>35	44.0 (40.5)	37.0 (40.0)	7.0 (43.8)		

AST (U/L)	15–45	63.0 (57.3)	57.0 (60.5)	6.0 (37.5)	0.16	0.08
	>45	47.0 (42.7)	37.0 (39.5)	10.0 (62.5)		

LDH (U/L)	≤500	45.0 (47.5)	40.0 (48.0)	5.0 (42.0)	0.04	0.67
	>500	50.0 (52.5)	43.0 (52.0)	7.0 (58.0)		

Albumin (g/dL)	≥3.5	13.0 (56.5)	11.0 (61.0)	2.0 (40.0)	0.17	0.62
	<3.5	10.0 (43.5)	7.0 (39.0)	3.0 (60.0)		

ESR (mm/h)	≤30	49.0 (51.5)	42.0 (52.5)	7.0 (47.0)	0.04	0.67
	>30	46.0 (48.5)	38.0 (47.5)	8.0 (53.0)		

D-dimer	≤200	13.0 (48.0)	11.0 (48.0)	2.0 (50.0)	0.01	0.94
	>200	14.0 (52.0)	12.0 (52.0)	2.0 (50.0)		

CPK (U/L)	≤200	67.0 (87.0)	61.0 (89.7)	6.0 (66.7)	0.22	0.05
	>200	10.0 (13.0)	7.0 (10.3)	3.0 (33.3)		

PTT (s)	24–36	41.0 (74.5)	31.0 (70.5)	10.0 (91.0)	0.19	0.16
	>36	14.0 (25.5)	13.0 (29.5)	1.0 (9.0)		

PT (s)	11–12	14.0 (25.5)	12.0 (27.3)	2.0 (18.0)	0.08	0.54
	>12	41.0 (74.5)	32.0 (72.7)	9.0 (82.0)		
INR	<1.5	19.0 (86.5)	14.0 (82.5)	5.0 (100)	0.21	0.31
	>1.5	3.0 (13.5)	3.0 (17.5)	0.0 (0.0)		

BS (mg/dL)	<60	2.0 (2.0)	2.0 (2.5)	0.0 (0.0)	0.06	0.83
	60–200	90.0 (92.0)	77.0 (91.5)	13.0 (93.0)		
	>200	6.0 (6.0)	5.0 (6.0)	1.0 (7.0)		

UARBC	≤4	27.0 (93.0)	21.0 (95.5)	6.0 (86.7)	0.16	0.38
	>4	2.0 (7.0)	1.0 (4.5)	1.0 (14.3)		

UAWBC	Normal	29.0 (88.0)	21.0 (84.0)	8.0 (100)	0.21	0.23
	Abnormal	4.0 (12.0)	4.0 (16.0)	0.0 (0.0)		

Frequency (%) was used for describing the categorical variables. The Cramer's V, chi-square, and Fisher exact tests were used to measure the association between categorical variables and status. The first-time measured values of all laboratory findings are summarized in categories. CR was divided into normal/abnormal categories based on age groups.

**Table 3:** Descriptive statistics of drugs being used for children with COVID-19 in Shahid Sadoughi Hospital in Yazd.

Variables		Total (*n* = 183)	Survived (*n* = 159)	Deceased (*n* = 24)	Cramer's V/Eta	*P* value
Kaletra	Yes	29.0 (15.8)	25.0 (15.7)	4.0 (16.7)	0.009	0.9
	No	154.0 (84.2)	134.0 (84.3)	20.0 (83.3)		

Hydroxychloroquine	Yes	23.0 (12.6)	19.0 (12.0)	4.0 (16.7)	0.05	0.52
	No	160.0 (87.4)	140.0 (88.0)	20.0 (83.3)		

Oseltamivir	Yes	11.0 (6.0)	8.0 (5.0)	3.0 (12.5)	0.10	0.15
	No	172.0 (94.0)	151.0 (95.0)	21.0 (87.5)		

Enoxaparin	Yes	27.0 (15.0)	24.0 (15.0)	3.0 (12.5)	0.025	0.74
	No	156.0 (85.0)	135.0 (85.0)	21.0 (87.5)		

Ribavirin tab	Yes	2.0 (1.0)	2.0 (1.3)	0.0 (0.0)	0.04	0.58
	No	181.0 (99.0)	157.0 (98.7)	24.0 (100)		

ASA	Yes	13.0 (7.0)	13.0 (8.0)	0.0 (0.0)	0.1	0.15
	No	170.0 (93.0)	146.0 (92.0)	24.0 (100)		

Remdesivir	Yes	53.0 (29.0)	45.0 (28.0)	8.0 (33.0)	0.04	0.61
	No	130.0 (71.0)	114.0 (72.0)	16.0 (67.0)		

Dexamethasone amp	Yes	34.0 (18.5)	29.0 (18.0)	5.0 (21.0)	0.02	0.76
	No	149.0 (81.5)	130.0 (82.0)	19.0 (79.0)		

Dexamethasone tab	Yes	2.0 (1.0)	2.0 (1.3)	0.0 (0.0)	0.04	0.58
	No	181.0 (99.0)	157.0 (98.7)	24.0 (100)		

Heparin	Yes	14.0 (8.0)	13.0 (8.0)	1.0 (4.0)	0.05	0.49
	No	169.0 (92.0)	146.0 (92.0)	23.0 (96.0)		

Interferon	Yes	14.0 (8.0)	12.0 (7.5)	2.0 (8.5)	0.01	0.89
	No	169.0 (92.0)	147.0 (92.5)	22.0 (91.5)		

Favipiravir	Yes	19.0 (10.5)	19.0 (12.0)	0.0 (0.0)	0.13	0.07
	No	164.0 (89.5)	140.0 (88.0)	24.0 (100)		

Vitamin D	Yes	17.0 (9.5)	16.0 (10.0)	1.0 (4.0)	0.07	0.35
	No	166.0 (90.5)	143.0 (90.0)	23.0 (96.0)		

Famotidine	Yes	27.0 (15.0)	26.0 (16.5)	1.0 (4.0)	0.12	0.18
	No	156.0 (85.0)	133.0 (83.5)	23.0 (96.0)		

Methylprednisolone	Yes	28.0 (15.0)	21.0 (13.0)	7.0 (29.0)	0.15	0.04
	No	155.0 (85.0)	138.0 (87.0)	17.0 (71.0)		

Intravenous immunoglobulin	Yes	6.0 (3.5)	5.0 (3.0)	1.0 (4.0)	0.02	0.79
	No	177.0 (96.5)	154.0 (97.0)	23.0 (96.0)		

Frequency (%) was used for describing the drugs being used. The Cramer's V, chi-square, and Fisher exact tests were used to measure the association between the drugs being used and status.

**Table 4:** Hazard ratio of characteristics of children with COVID-19 in Shahid Sadoughi Hospital in Yazd.

Variables		Univariate		Multivariate	
		HR (95% CI)	*P* value	HR, (95% CI)	*P* value
Age	1 month–1 year	1.31 (0.42–4.08)	0.63	0.00	0.98
	1 year–5 years	0.83 (0.29–2.39)	0.73	0.73 (0.12–4.36)	0.72
	5 years–13 years	0.40 (0.11–1.43)	0.16	0.58 (0.08–4.22)	0.59
	13 years–18 years	—	—	—	—

Sex	Boys	—	0.72	—	0.22
	Girls	1.16 (0.52–2.58)		0.43 (0.11–1.66)	

History of hospitalization	No	—	0.007	—	0.09
	Yes	3.0 (1.35–6.7)		3.63 (0.78–16.82)	

Underlying diseases	No	—	0.16		0.29
	Yes	1.78 (0.79–4.0)		1.2 (0.55–7.3)	

CT findings	Normal	—	0.18		
	Abnormal	0.24 (0.03–1.88)		—	

O_2_ saturation without support	≥94%	—	0.04	—	0.15
	<94%	3.03 (1.05–8.73)		2.85 (0.68–11.98)	

Lymphs (%)	No	—	0.08		0.61
	Yes	2.23 (0.9–5.5)		0.7 (0.18–2.74)	

AST (U/L)	15–45	—	0.09		
	>45	2.37 (0.86–6.52)			

CPK (U/L)	≤200	—	0.07		
	>200	3.62 (0.9–14.5)			

PICU admission	No	—	<0.001		
	Yes	27.24 (6.4–115.93)			

Intubation	No	—	<0.001		
	Yes	14.3 (6.04–33.86)			

Ventilator support	No		<0.001		
	Yes	16.08 (6.87–35.85)			

Length of hospital stay (day)	≤7	—	—	—	—
	8–14	3.27 (1.33–8.0)	0.01	0.37 (0.04–3.45)	0.39
	≥15	3.77 (1.21–11.7)	0.02	1.68 (0.24–11.85)	0.6

**Table 5:** Posterior estimation of the hazard ratio of characteristics of children with COVID-19 in Shahid Sadoughi Hospital in Yazd.

Variables		Mean	Std. dev	95% HPD	HR (95% HPD)
Age	1 month–1 year	−13.26	2.06	(−17.27, 9.83)	0.00
	1 year–5 years	−0.23	0.08	(−0.34, 0.01)	0.79 (0.71, 1)
	5 years–13 years	−0.47	0.15	(−0.79, 0.29)	0.62 (0.45, 075)
	13 years–18 years				

Sex	Boys		0.14		
	Girls	−0.63		(−0.93, −0.43)	0.53 (0.39, 0.56)

History of hospitalization	No				2.77 (2.14, 3.78)
	Yes	1.02	0.16	(0.76, 1.33)	

Underlying diseases	No		0.1		1.75 (1.46, 2.16)
	Yes	0.56		(0.38, 0.77)	

O_2_ saturation without support	≥94%		0.14		2.29 (1.78, 3.03)
	<94%	0.83		(0.58, 1.11)	

Lymphopenia	No	0.24	0.04	(0.17, 0.31)	1.27 (1.18, 1.36)
	Yes				

Length of hospital stay (day)	≤7				
	8–14	−0.79	0.12	(−1.02, −0.58)	0.45 (0.36, 0.56)
	≥15	0.64	0.14	(0.46, 0.97)	1.89 (1.58, 2.64)

## Data Availability

The registered data used to provide the findings of this study are restricted by the Ethics Committee of Shahid Sadoughi University of Medical Sciences and Health Services in order to protect patient privacy. Data are available from Mehran Karimi at drmehrankarimi@yahoo.com for researchers who meet the criteria for access to confidential data.
